# Clinical trials in rare diseases: a review of practice

**DOI:** 10.1186/1745-6215-14-S1-P34

**Published:** 2013-11-29

**Authors:** Stuart Bell, Paula Williamson, Simon Day, Keith Wheatley, John Whitehead, Catrin Tudur Smith

**Affiliations:** 1 University of Liverpool, Liverpool, UK; 2 Roche Products Limited, Welwyn Garden City, UK; 3 Lancaster University, Lancaster, UK; 4 Birmingham University, Birmingham, UK

## Background

The evaluation of treatments for rare diseases presents a number of challenges for trial practitioners, regulators and policy makers. Small sample sizes mean that ‘standard' approaches to trial design and analysis may not be appropriate and alternatives such as Bayesian trial designs have been recommended [Lilford et al 1995]. However, little is known about the design and analysis approaches that have been implemented in practice.

## Methods

We will perform a review of rare disease clinical trials to

(i) describe the trial design characteristics that have been applied to rare disease clinical trials;

(ii) summarise the characteristics that facilitate or hinder the design, conduct and publication of rare disease clinical trials;

(iii) summarise approaches in which researchers have attempted to improve the efficiency of rare disease clinical trials;

(iv) identify whether alternative design and analysis considerations may have been possible in a given trial.

Rare disease clinical trials will be identified through electronic search of trial registries (http://www.clinicaltrials.gov and http://www.orpha.net). Trials will be assessed for inclusion by two independent reviewers and relevant data extracted. Trials will be summarised qualitatively with regard to the methodologies adopted and their strengths and weaknesses.

## Results

Preliminary results will be available to discuss at the conference.

## Conclusions

The purpose of this review is to explore how rare disease trials have been implemented. This will provide a much needed summary of approaches that have been used in practice, highlighting where possible, why particular designs have been chosen.

